# Operability of a Resonance-Based Viscoelastic Haemostatic Analyzer in the High-Vibration Environment of Air Medical Transport

**DOI:** 10.3390/jcm11133630

**Published:** 2022-06-23

**Authors:** Johannes Zipperle, Bernhard Ziegler, Herbert Schöchl, Wolfgang Voelckel, Christoph J. Schlimp, Daniel Oberladstätter

**Affiliations:** 1 Ludwig Boltzmann Institute for Traumatology, The Research Center in Cooperation with AUVA, 1200 Vienna, Austria; johannes.zipperle@trauma.lbg.ac.at (J.Z.); herbert.schoechl@medical-education.at (H.S.); christoph.schlimp@trauma.lbg.ac.at (C.J.S.); 2 Perioperative Medicine and General Intensive Care Medicine, Department of Anaesthesiology, Paracelsus Medical University, 5020 Salzburg, Austria; b.ziegler@salk.at; 3 ÖAMTC Air Rescue, 1030 Vienna, Austria; wolfgang.voelckel@auva.at; 4 Department of Anaesthesiology and Intensive Care Medicine, AUVA Trauma Centre Salzburg, Academic Teaching Hospital of the Paracelsus Medical University, 5020 Salzburg, Austria; 5 Department of Anaesthesiology and Intensive Care Medicine, AUVA Trauma Centre Linz, 4010 Linz, Austria

**Keywords:** viscoelastic tests, point-of-care, emergency helicopter, trauma-induced coagulopathy, haemostasis

## Abstract

Trauma and bleeding are associated with a high mortality, and most of these deaths occur early after injury. Viscoelastic haemostatic tests have gained increasing importance in goal-directed transfusion and bleeding management. A new generation of small-sized and thus portable ultrasound-based viscoelastic analysers have been introduced in clinical practice. We questioned whether a promising candidate can be used in emergency helicopters, with a focus on the susceptibility to vibration stress. We investigated whether the high vibration environment of an emergency helicopter would affect the operability of an ultrasound-based viscoelastic analyser and would yield reproducible results in flight and on the ground. We drew blood from 27 healthy volunteers and performed simultaneous analyses on two TEG 6s. Each measurement was performed in-flight on board an Airbus H135 emergency helicopter and was repeated on the ground, close to the flight area. Results from both measurements were compared, and the recorded tracings and numeric results were analysed for artifacts. Vibratometric measurements were performed throughout the flight in order to quantify changes in the magnitude and character of vibrations in different phases of helicopter operation. The high vibration environment was associated with the presence of artifacts in all recorded tracings. There were significant differences in citrated Kaolin + Heparinase measurements in-flight and on the ground. All other assays increased in variability but did not show significant differences between the two time points. We observed numerous artifacts in viscoelastic measurements that were performed in flight. Some parameters that were obtained from the same sample showed significant differences between in-flight and on-ground measurements. Performing resonance-based viscoelastic tests in helicopter medical service is prone to artifacts. However, a 10 min delay between initiation of measurement and take-off might produce more reliable results.

## 1. Introduction

Trauma represents a predominant cause of death worldwide. The majority of these deaths are due to bleeding and occur in the first hours after injury [1]. About one-third of severely injured patients present with trauma-induced coagulopathy (TIC), a multifactorial bleeding tendency that is associated with a poor outcome. TIC is characterized by the loss of functional coagulation factors, activation of the protein C pathway, platelet dysfunction, and fibrinolytic activation [2,3]. Standard laboratory coagulation tests have proven insufficient to guide clinical decision-making in the treatment of TIC [4]. In contrast, viscoelastic haemostatic assays (VHA) are performed in whole blood and provide information on the contribution of coagulation factors and platelets to clot strength as well as on the magnitude of fibrinolytic activation or shutdown [5,6,7,8]. Viscoelastic haemostatic testing has laid the groundwork for effective blood product and transfusion management [9]. VHAs have therefore gained considerable importance in goal-directed haemostatic therapy and bleeding management. The classic operating principle of VHAs is based on rotating parts measuring the elastic shear modulus of whole blood between two surfaces as the blood clot develops, and this is traced as a curve over time [10].

The initial principle of a viscoelastic test was first described by Hartert in 1948 [11]. This initial device was highly shock-sensitive and therefore replaced over the years by more robust analysers such as ROTEM (rotational thrombelastometry) or TEG (thrombelastography). A number of manufacturers have adapted and modified this principle for modern VHAs, which has since been established as a gold standard in POC bleeding diagnostics [12]. A major prerequisite for POC technology is the operability of the device next to the patient. In trauma critical care and elective surgery, this also means that the device has to be handled under stressful conditions and by multiple operators. An essential advantage of novel, resonance-based VHAs is the extent of automation and the emphasis on user experience. One of the new generations of POC devices for haemostaseology is the Haemonetics TEG6s, a promising candidate for employment in a mobile area of application. 

The new resonance-based TEG6s hemostasis analyzer (Haemonetics^®^, Boston, MA, USA) was introduced to eliminate reagent handling and minimise pipetting errors, thereby reducing inter- and intra-operator variability. It employs a single-use microfluidic cartridge to minimize sample volume and uses a resonance-based operating principle. The blood sample is exposed to vibration at a fixed frequency range (20–500 Hz) and starts to resonate. Clot strength is then assessed by measuring the frequency of resonance, and a familiar thrombelastography tracing is generated by the operating system [13]. Clinical evaluation has demonstrated high reliability and reproducibility when comparing resonance-based devices with conventional VHA technology [13,14,15], while other researchers found only moderate correlation [16,17]. The inter-device reliability of TEG6S was observed to be very high [18]. Aside from that, the TEG6s measurement principle, miniaturization of design, and sample volume, as well as an intuitive user interface facilitate the operation of the device under the exposed conditions of military settings and air medical transport. These features would not only enable the pre-clinical assessment of haemostasis in emergency evacuation, but could also allow for the monitoring of blood coagulation during long-term, secondary critical care transport. That way, pre- or inter-hospital coagulation management could be improved. Indeed, initial experiments yielded promising results when the TEG6s was exposed to shaking and sudden strikes during measurements. Several experimental and one prehospital inflight study revealed conflicting results [19,20,21,22]. In order to evaluate in detail the factors that potentially influence the analytical quality of TEG6s during in-flight missions, we compared test results during helicopter operations with results that were obtained on the ground. The aim of the study was to test the usability and reliability of TEG6s during helicopter air medical transport.

## 2. Materials and Methods

### 2.1. Study Design

This study was approved by the local ethics committee (AUVA EK 23/2019) and was conducted during scheduled HEMS crew member training missions in 2019 and 2020. We designed and conducted a two-armed prospective observational study in an attempt to investigate the effects of a high vibration environment on viscoelastic test results. In one arm of the study, we placed two TEG 6s analysers in an EMS helicopter (H135, Airbus Helicopters SAS^®^, Marignane, France). Time from initiation of measurement on the ground to take-off was recorded. In the other arm, we repeated the measurement using the same citrated blood tubes under solid ground conditions close to the flight area. Finally, results from both measurements were compared and traces were evaluated for reproducibility and the presence of artifacts.

### 2.2. Viscoelastic Testing

**Figure 1:**
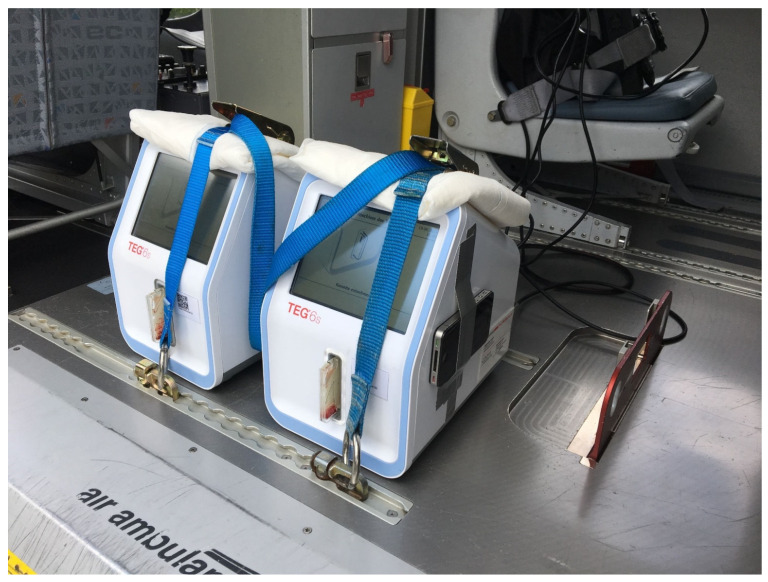
TEG6s devices mounted on the helicopter floor. In contrast to the picture, the device for vibratometric measurement was fixed to the floor for study measurements.

**Figure 2:**
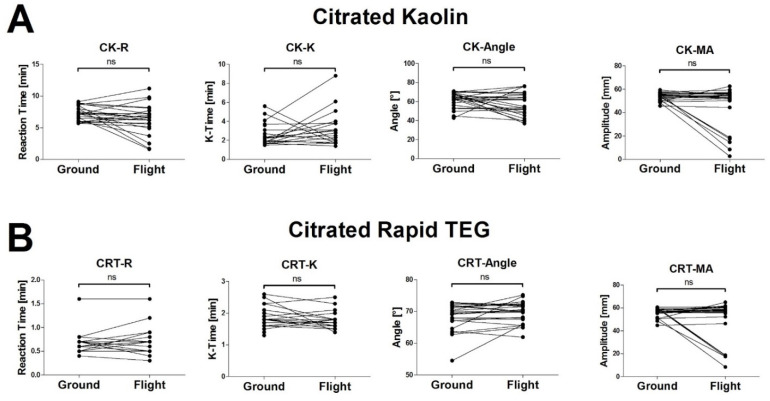

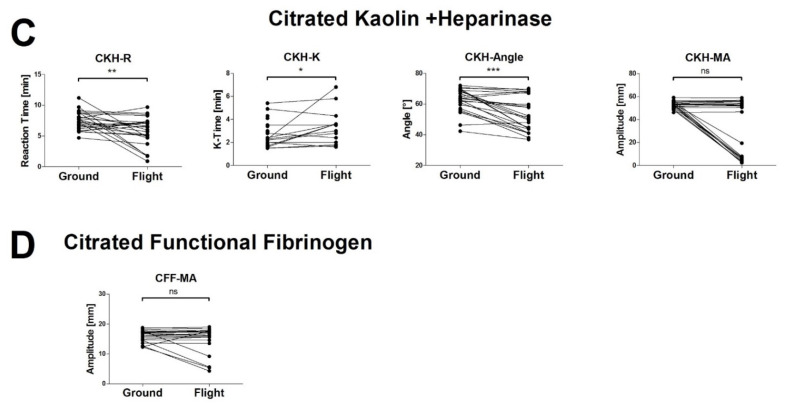
Viscoelastic haemostatic measurements from healthy volunteers on the ground and in-flight. Corresponding results from the same donor are connected with a line. Paired *t*-tests were performed to assess differences between both measurement conditions. Note that in many cases, statistical analysis returned no significant differences between time points. However, when looking on individual data points, measurements yielded different results in-flight and on the ground. CK, Citrated Kaolin; CRT, Citrated Rapid TEG; CKH, Citrated Kaolin + Heparinase; CFF, Citrated Functional Fibrinogen; * *p* < 0.05; ** *p* < 0.01; *** *p* < 0.005; ns, not significant.

**Figure 3:**
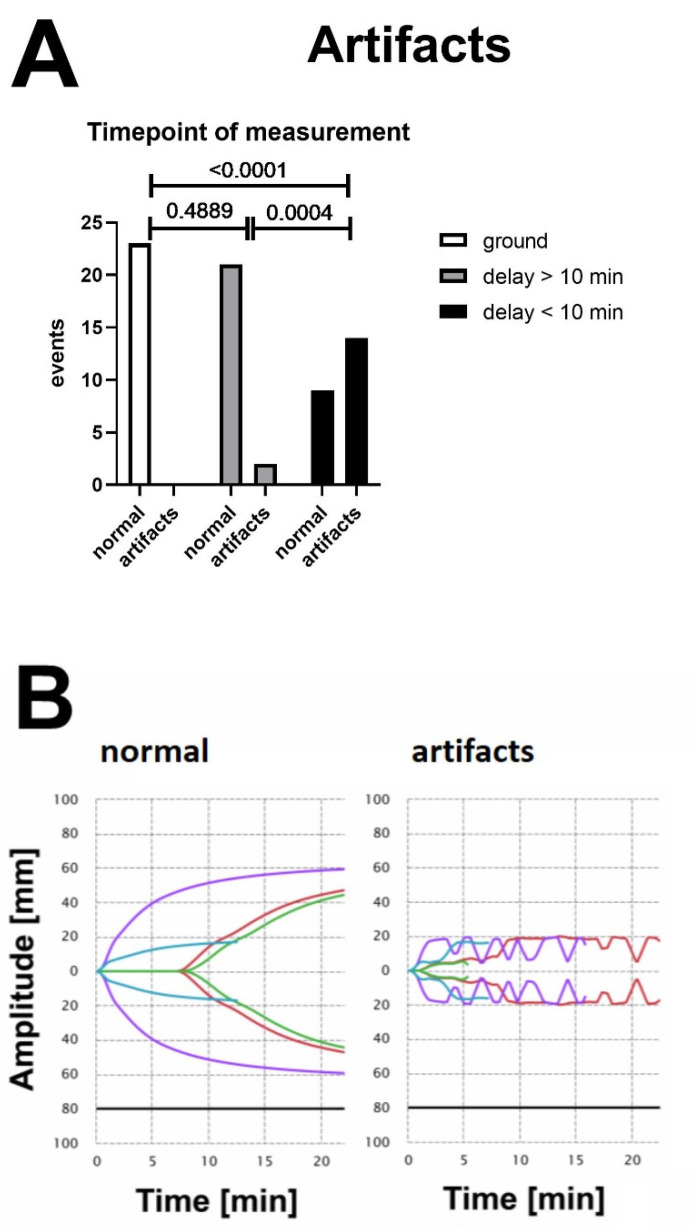
(**A**) Frequency of occurrence of artifacts in the tracings of TEG6s measurements. Unlabelled curves were inspected by an operator, who was blinded to the experiment. Data were then stratified by the time from beginning of measurement until the engine was started (“delay < 10 min vs. >10 min”). No artifacts were found in any of the ground measurements. When the measurement was started within 10 min prior to starting the engine, a high number of artifacts were present. In case a certain clot firmness was reached when the engine was started (delay > 10 min), susceptibility of the measurement to artifacts was reduced. Depicted *p*-values are derived from the applied Chi-square test (**B**). Representative tracings of measurements on the ground and in-flight (engine on). Multiple inflections and artifacts are visible in measurements that were started immediately (delay < 10 min).

After obtaining informed consent, we drew 2.7 mL blood from volunteers into tubes containing 0.3 mL of buffered trisodium citrate 3.2% (S-Monovette^®^; Sarstedt AG, Nürmbrecht, Germany), with a citrate:blood volume ratio of 1:9. None of the volunteers had any history of coagulopathy or anticoagulant medication within the last two weeks. All blood samples were drawn under minimal stasis and were processed within two hours [23]. Samples were analysed on two TEG6s devices (Haemonetics^®^, Boston, MA, USA) using a cartridge with four different assays, namely (1) Kaolin—activating intrinsic pathway, (2) Kaolin + Heparinase—in combination with Kaolin test, indicating the presence of heparin or heparin-like substances, (3) RapidTEG—additional fast activation of the extrinsic pathway, and (4) Functional Fibrinogen—to assess fibrinogen contribution to clot strength (Global Hemostasis-Assay, Haemonetics Cat. Nr. 07-601, Citrated: K, KH, RT, FF. In contrast to the cartridges available in the US, this cartridge does include the CK.LY30 parameter). Two samples were run on the two TEG 6s devices simultaneously in the helicopter (Figure 1). The prime viscoelastic parameters reaction time (R, time until initiation of clotting), K-time (K, time until a certain amplitude is reached), α-angle (slope of the emerging viscoelastic tracing), and maximum amplitude (MA, amplitude of the generated curve) are given for the assays CK (Citrated Kaolin), CRT (Citrated Rapid TEG), and CKH (Citrated Kaolin + Heparinase) (Figure 2A–C). Maximum amplitude is the only given parameter for CFF (Citrated Functional Fibrinogen) (Figure 2D). Measurement was initiated before take-off (helicopter on ground) while the engines were off or in ground-idle state. During measurement, no extreme flight manoeuvres (e.g., emergency landing) were performed. After the measurements were finished, both devices were transferred out of the helicopter onto solid ground, and the samples were run again. Test results from ground measurements were compared with respective in-flight tests. Tests were run according to manufacturer’s instructions until maximum amplitude (MA) was confirmed in all assays. We performed in-flight testing at an outside temperature between 9° and 21° Celsius and at an altitude between 1170 and 2030 m above sea level. VHA tracings from the ground and helicopter setting were visually assessed for artifacts by a blinded operator (Figure 3B). An artifact was defined as at least one artificial inflection in the tracing that was incompatible with natural clotting dynamics.

### 2.3. Vibratometric Measurements

**Figure 4:**
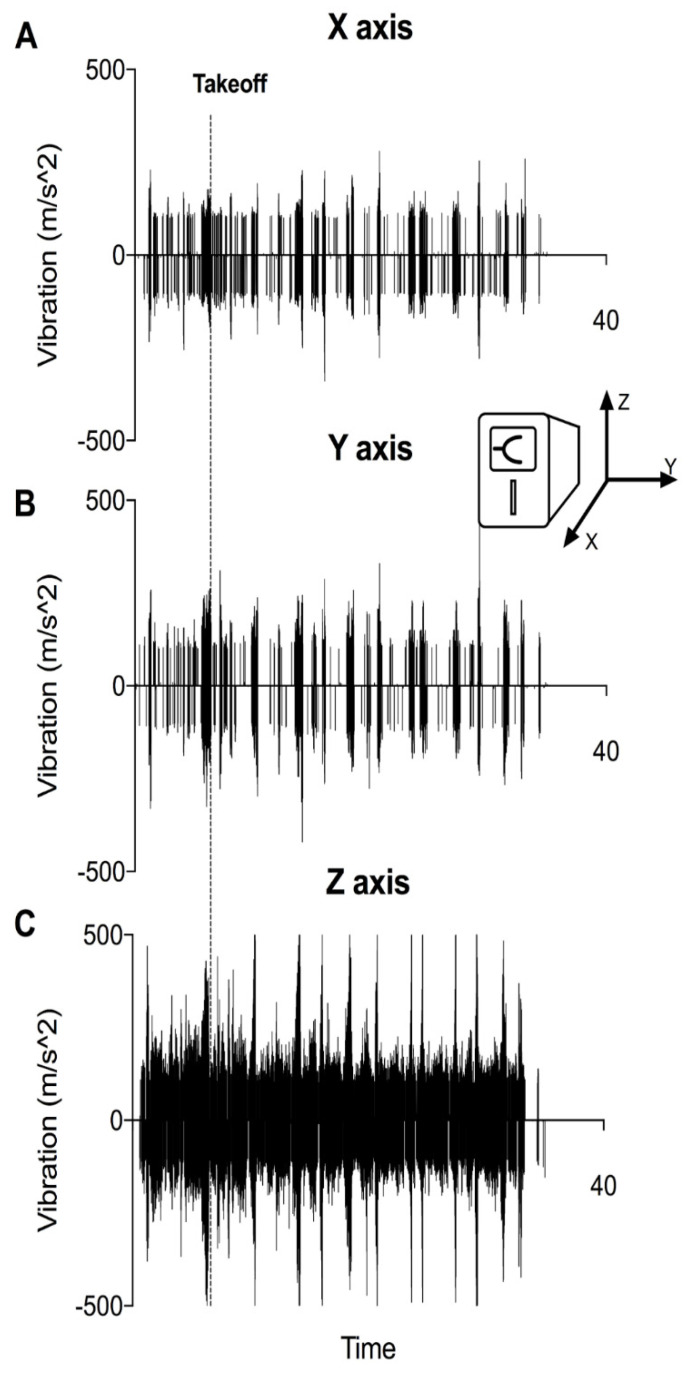
(**A**−**C**) Representative vibratometric measurement within the high-vibration environment of air medical transport. A smartphone with an accelometric sensor and a respective mobile application was directly attached to the same surface on which both viscoelastic analysers were placed. Vibration is given as acceleration in m/s^2^. Alignment of the phone and arising axes with regard to both VHAs is illustrated in the insert. Measurement was initiated when the engine of the aircraft was started. Time of take-off is marked with a dashed line. Vibratometric measurements indicate that the devices are already exposed to a high-vibration environment prior to take-off as soon as the engine is started, and that the majority of vibrations occur on the *Z*-axis. (**A**) Vibration at *X*-axis, (**B**) Vibration at *Y*-axis and (**C**) Vibration at *Z*-axis.

Magnitude and timing of vibrations were quantified with a mobile vibrometer application for iPhone IOS (Apple Inc., Cupertino, CA, USA), using accelerometric data. The device (iPhone 5, Apple Inc., Cupertino, CA, USA) was fixed directly to the aircraft floor and was aligned to the two TEG6s devices (directions and axes are illustrated in the Figure 4 insert). The devices were placed in the helicopter in a way that the *Y*-axis corresponds to the longitudinal axis of the helicopter, the *X*-axis to the short axis, and the Z axis to the vertical axis (see also Figure 1).

### 2.4. Statistical Analysis

Data were extracted from the devices and collected in Microsoft Excel (Microsoft Corp., Redmond, WA, USA). Visualisation and statistical analysis of data were then carried out in GraphPad Prism 5 (Prism Software, San Diego, CA, USA). Data points from ground were compared with respective in-flight measurements. All datasets were checked for normal distribution with a Shapiro–Wilk test and were analysed via paired t-test or Wilcoxon signed rank test, where applicable. The contingency of normal versus artifact tracing events was calculated using a Chi-square test. A *p* value < 0.05 was considered significant.

## 3. Results

### 3.1. VHAs Are Exposed to Vibration as Soon as the Engine Is Started

Vibratometric measurements revealed that detectable vibrations are present even before take-off (Figure 4A–C). While during ground-idle state, vibrations were mild and homogeneous, they showed a strong increase during take-off. Other regular flight procedures also showed a relevant peak in vibration. Upon distinction of alignment of vibration, intensity appeared most prominent in the *Z*-axis (Figure 4C).

### 3.2. The High Vibration Environment of Helicopter Operations Alters VHA Results

In CK and CRT assays, p revealed no significant differences between datasets that were obtained on the ground and in-flight. However, individual values in the MA measurements were fundamentally different in-flight and on the ground (Figure 2A,B). In the CKH assay, there were significant differences between ground and in-flight measurements (Figure 2C). With the assessment of functional fibrinogen (CFF-MA), p again did not show significant differences in-flight and on the ground, although 5 out of 24 measurements showed fundamentally different results (Figure 2D). The CK.LY30 Parameter was out of range in three measurements (values 3.0, 4.6 and 9.0%; all in-flight).

### 3.3. Vibration-Induced Artifacts Are Associated with Unreliable Test Results in VHAs

No artifacts occurred in measurements that were performed on the ground and outside of the aircraft (Figure 3A, ground). Measurements that were performed on-board were stratified by the time the test was running before take-off. When the assay was running for longer than 10 min prior to take-off, only few artifacts were present (Figure 3A, delay > 10 min). The majority of inflections occurred on measurements that were started immediately before the aircraft took off (Figure 3A, delay < 10 min). A representative illustration of normal and artifact tracings is given in Figure 3B.

## 4. Discussion

The results of the current study revealed that initiating viscoelastic coagulation measurement on the TEG6s immediately prior to air medical transport in a helicopter did not provide reliable test results for clinical decision making. These findings should also be true for starting measurements during helicopter transport, as several regular flight manoeuvres showed a peak in vibration. TEG6s parameters are severely affected by the high-vibration environment of helicopters in operations. However, starting a measurement >10 min before take-off could be considered suitable for clinical decision-making.

Although there were no significant differences between the MA measurements from the ground and in flight, individual data points displayed a dramatic reduction in amplitude. Since both study samples were obtained from healthy volunteers and were pipetted from the same tube, a considerable reduction in amplitude from one time point to another could have been falsely interpreted as a massive loss of coagulation capacity [24]. Other parameters in the Citrated Kaolin + Heparinase assay (CKH) displayed a significant difference between ground and in-flight measurements. We cannot refute the assumption that the distance from floor-mounting to the level of the specific test in the cartridge/analyser will have impact on the amount of vibration to which the clot is exposed; thus, different intensity of vibration might apply to the specific tests.

In an attempt to assess the operability of the device under helicopter-like conditions, Scott et al. [20] tested TEG6s control cartridges in a rotary wing aircraft simulator and found that the majority of values were in the normal range. In contrast to our measurements, the vibrations in the simulator may not sufficiently reflect the vibration reality in the helicopter. This might be the reason why their results only showed relevant alterations in extreme flight manoeuvres. Boyé et al. [21] did not find significant changes in results due to heat or vibration (simulated by a shaker, 100 Hz, but saw differences in MA, α-angle, and especially functional fibrinogen (CFF) due to the change in altitude simulated in a hypobaric chamber. The constant shaking at 100 Hz does not adequately reflect vibration characteristics of a helicopter during take-off, in-flight manoeuvres, or landing. However, when testing the analyser in actual flight operations with whole blood from healthy volunteers, Bates et al. [22] found a poor correlation between measurements on the ground and in flight, which corresponds well with our findings. Based on our results, we do not recommend the un-cushioned operation of resonance-based VHAs in the high-vibration environment of air medical transport in order to guide clinical decision-making. The TEG6s only delivered reliable test results when clot firmness already exceeded a certain threshold level prior to take-off. This is in line with McGill’s observations, who placed a TEG6s on a platelet agitator and initiated agitation and measurements simultaneously [25]. Tracings in this study resembled our results when measurements were started during ground-idle immediately before take-off. These findings suggest that extensive external movement (such as vibration) interferes with the technical ability of TEG6s to determine clot stiffness mainly in the early but not in the later phase of measurement. 

Our test setup included the direct mounting of the devices to the aircraft cabin floor in accordance with the operator’s flight safety regulations for medical equipment and was signed off by both the Flight Operations Manager and head of technology department. This setup would eventually also result in the direct transmission of vibrations onto the TEG6s device. We suggest that the further evaluation of resonance-based VHAs under vibration-exposed conditions should conducted in a cushioned setup (e.g., embedded in a casing) that would allow for the absorbance of vibration and shocks. 

On the assumption that (I) early administration of blood products during air medical transport might gain further importance (PAMPer Trial) [26], (II) goal-directed management of major haemorrhage with coagulation factor concentrates might be favourable over plasma transfusions (RETIC) [27], and (III) the pre-hospital administration of fibrinogen concentrate to treat hypofibrinogenaemia will eventually play a role in trauma critical care (FIinTIC) [28], the operation of mobile VHAs in emergency service vehicles might represent a favourable development.

## 5. Limitations

We conducted a prospective observational study analysing blood from healthy volunteers on a VHA aboard a rotary wing aircraft. We did not test the device under fixed-wing flight conditions. Since we worked with blood samples from healthy donors, we could not observe a broader, pathophysiological spectrum of parameters. However, the results were so inconsistent that we are unlikely to obtain different results from blood samples gathered from coagulopathic patients. Furthermore, our study was conducted with only one model of resonance-based VHAs, namely Haemonetics TEG6s. Despite being relevant for practical use, for not impacting the HEMS crew training, none of our measurements were initiated when the aircraft was already in the air. So, the results might be different when the VHA is used only in level flight, without a take-off phase the during measurement period.

## 6. Conclusions

The TEG6s represents an easy-to-use VHA that enables multiple simultaneous measurements using only minimal amounts of blood and provides a user-friendly experience. Initiating viscoelastic coagulation measurement on the TEG6s during air medical transport in a helicopter does not seem feasible due to the high-vibration artifacts. However, starting a measurement 10 min before turning on helicopter engines or take-off might be considered an interesting option for future studies and clinical decision-making with this device in critical situations where starting coagulation management during flight operation is considered.

## Data Availability

The data presented in this study are available on request from the corresponding author.
